# Intrusions of a drowsy mind: neural markers of phenomenological unpredictability

**DOI:** 10.3389/fpsyg.2015.00202

**Published:** 2015-03-12

**Authors:** Valdas Noreika, Andrés Canales-Johnson, Justin Koh, Mae Taylor, Irving Massey, Tristan A. Bekinschtein

**Affiliations:** ^1^Cognition and Brain Sciences Unit, Medical Research CouncilCambridge, UK; ^2^Department of Psychology, University of CambridgeCambridge, UK; ^3^Laboratory of Cognitive and Social Neuroscience, Universidad Diego PortalesSantiago, Chile; ^4^Department of Medicine, University of CambridgeCambridge, UK; ^5^Hills Road Sixth Form CollegeCambridge, UK; ^6^Departments of English and Comparative Literature, University at BuffaloBuffalo, NY, USA

**Keywords:** consciousness, drowsiness, hypnagogic experiences, linguistic intrusions, perceptual imagery, EEG Dimension of Activation, EEG spectral power, Hori stages of sleep onset

## Abstract

The transition from a relaxed to a drowsy state of mind is often accompanied by hypnagogic experiences: most commonly, perceptual imagery, but also linguistic intrusions, i.e., the sudden emergence of unpredictable anomalies in the stream of inner speech. This study has sought to describe the contents of such intrusions, to verify their association with the progression of sleep onset, and to investigate the electroencephalographic processes associated with linguistic intrusions as opposed to more common hypnagogic perceptual imagery. A single participant attended 10 experimental sessions in the EEG laboratory, where he was allowed to drift into a drowsy state of mind, while maintaining metacognition of his own experiences. Once a linguistic intrusion or a noticeable perceptual image occurred, the participant pressed a button and reported it verbally. An increase in the EEG-defined depth of drowsiness as assessed by the Hori system of sleep onset was observed in the last 20 s before a button press. Likewise, EEG Dimension of Activation values decreased before the button press, indicating that the occurrence of cognitively incongruous experiences coincides with the rapid change of EEG predictability patterns. EEG hemispheric asymmetry analysis showed that linguistic intrusions had a higher alpha and gamma power in the left hemisphere electrodes, whereas perceptual imagery reports were associated with a higher beta power over the right hemisphere. These findings indicate that the modality as well as the incongruence of drowsiness-related hypnagogic experiences is strongly associated with distinct EEG signatures in this participant. Sleep onset may provide a unique possibility to study the neural mechanisms accompanying the fragmentation of the stream of consciousness in healthy individuals.

## INTRODUCTION

Temporal and spatial integration of experiences is a fundamental property of the stream of consciousness (James, 1890), which seems to hold not only for the perception of relatively stable properties in the physical world, but also for internally generated experiences, such as inner speech. Departures from this principle are often, as in the case of schizophrenia (Vogeley and Kupke, 2007), regarded as pathological; such events, however, may also occur as ‘mind pops’ during waking hours within the normal population (Elua et al., 2012). A particularly wide range of behavioral, perceptual, and cognitive alterations that lead to the fragmentation of consciousness seems to take place in the transition between wakefulness and sleep (Goupil and Bekinschtein, 2011), and – as we show here – they can be successfully studied in a drowsy participant in the sleep lab.

Subjective experiences during this transition are sometimes referred to as *hypnagogia* (Mavromatis, 1987), with *hypnagogic imagery* being the most paradigmatic alteration of consciousness during sleep onset (McKellar and Simpson, 1954). The complexity and intensity of hypnagogic imagery can range from vague and fleeting impressions to fully formed images or even hallucinated dreams (Foulkes et al., 1966; Nielsen, 1995), and the most common forms of hypnagogic images are visual, auditory, and bodily experiences (Foulkes and Vogel, 1965). The occurrence of hypnagogic imagery experiences, both visual and kinaesthetic, were linked by Germain and Nielsen (2001) to a decrease of theta, alpha, and beta power in a time window preceding their report. In addition to hypnagogic imagery, other types of hypnagogia may include awareness of sleep onset (Kaplan et al., 2007), distorted perception of space (Bareham et al., 2014), and time (Minkwitz et al., 2012), as well as linguistic alterations such as those we describe below.

In the spring of 2011, Massey (who subsequently joined our experiment), approached Bekinschtein, to help him understand a phenomenon he had observed while he himself was falling asleep. Massey had noticed that, during that transitional process, words or phrases sometimes arose in his mind that had nothing to do with the imagery that was present in his thoughts at the time. In other words, there was a divergence or dissociation of the language stream from the other content of the incipient dream. It was Massey’s hope that some light might be cast on this odd phenomenon, which he described as *linguistic intrusions*, by a study of the processes taking place in the brain during hypnagogia. Being fully aware that his own experience might be entirely idiosyncratic, and might therefore be of limited interest to science, Massey (2009, pp. 65–70) reviewed much of the relevant literature, and found that the phenomenon he had noticed had, in fact, been widely reported, but never studied experimentally.

As it happens, the perfect example, which has since become famous, occurs not in a scientific but in a literary text, though there is also abundant material in scientific publications (see below). In *Speak, Memory* Nabokov (1966, p. 33; whom, coincidentally, Massey had once served as an assistant at Harvard) writes: “Just before falling asleep, I often become aware of a kind of one-sided conversation going on in my mind, quite independently from the natural train of my thoughts. It is a neutral, detached, anonymous voice, which I catch saying words of no importance to me whatever—an English or a Russian sentence, not even addressed to me, and so trivial that I hardly dare give samples, lest the flatness I wish to convey be marred by a molehill of sense.” It was thought by Massey that any understanding of such linguistic intrusions that could be achieved by a focused experiment would help to elucidate the process behind such an event, not only in the case of Massey (1987, 2009), but in numerous other similar reports of hypnagogic and dream experiences, from both technical and literary sources (Kraepelin, 1906; Breton, 1962; Oswald, 1962; Arkin, 1981; Foulkes, 1985; Kiš, 1990).

Indeed, verbal reports of linguistic intrusions collected by Massey pointed to a sudden and unexpected alteration of the stream of consciousness in the linguistic domain: unpredictable words were appearing out of the context, sometimes ungrammatical or as a mixture of different languages. These reports seemed to be phenomenologically very different from typical instances of hypnagogic imagery that reflect changes of consciousness in the perceptual domain. As such, linguistic intrusions seemed to belong to a unique category of experiences, distinct not only from hypnagogic imagery, but also from hypnagogic auditory hallucinations (Jones et al., 2010), as a subject experiencing linguistic intrusions does not perceive them as external “voices”. For exemplary reports of linguistic intrusions and hypnagogic imagery, collected in the present study, see **Table 1**.

**Table 1:** Reports of linguistic intrusions and perceptual imagery.

Types of hypnagogia	Reports
Linguistic intrusions	The name **Gin He Song** came across my mind while I was thinking of admissions to the conference. It’s a familiar name but I don’t remember where I heard it, probably on the news.
	**Until they you I married**, some sort of family situation.
	The phrase **many dead line**, having something to do with trying to fall asleep.
	The phrase **learning to consume consciously from a master**, in the context of thinking about trying to fall asleep.
	I was trying to think of a name for a cocktail which had ‘Adam and Eve’ in it, and the word **Bobsled** came up quite inappropriately.

Perceptual imagery	Visual image of a *cross severed at an angle* in the context of some argument I was having.
	A visual image of *someone with an ivory collar being martyred by the church.*
	This is more in the way of a visual image, of *a devil squeezing a jam bottle so that the glass actually, actually is pushed inward,* but no verbal accompaniment.
	Visual image of *a curled up music manuscript,* entirely out of context.
	A slight visual image of *orange juice* while I was trying to put myself to sleep with a Russian cradle song.

Mixed content	**Equalizer oxygen**, complicated background, *a needle going through a piece of foam*.
	[**?Mengebee] attachment**, a phrase which seemed to have to do with *a man visitor introducing his wife, or not introducing her.*
	Nonsense word **Leeshoe** came up while I was imagining some sort of *overhanging canopy*.
	**Winter white man’s coat**, in the context of *putting a horse into a sort of violin case and zipping it up*.
	Something about the word **information** in the context of something concerning an *old woman and untying my boots*.

*Linguistic content is marked in bold, whereas perceptual content is marked in italic*.

With these subjective observations and theoretical considerations in mind, we carried out an exploratory case study of Massey, aiming to verify the hypnagogic status of linguistic intrusions, and to describe EEG processes associated with their occurrence. The following hypotheses were formulated:

### HYPOTHESIS 1

Following the self-observation of our participant Massey as well as subjective reports from other sources (Kraepelin, 1906; Breton, 1962; Oswald, 1962; Arkin, 1981; Foulkes, 1985; Kiš, 1990), we hypothesized that linguistic intrusions will occur during a period of increased drowsiness, placing them along the same axis of sleep onset as hypnagogic perceptual imagery (Hayashi et al., 1999; Germain and Nielsen, 2001).

### HYPOTHESIS 2

Following a previous study that associated hypnagogic imagery with distinct EEG spectral power changes, namely a decrease of theta to beta power in a time window preceding a report (Germain and Nielsen, 2001), we expected that linguistic intrusions would also show a change in the EEG spectral power in a time window preceding their report. There was not enough relevant literature to generate a more exact hypothesis regarding specific EEG frequency bands.

### HYPOTHESIS 3

Assuming that linguistic intrusions represent a type of alteration qualitatively different from that of hypnagogic imagery, we expected them to be associated with different patterns of EEG activity. In particular, given the well-established hemispheric asymmetry of linguistic processing (Ojemann et al., 1989; Vigneau et al., 2006), we expected linguistic intrusions to be associated with a higher EEG power in the left-hemisphere in the right-handed participant. There was no, however, relevant literature found to formulate a more exact hypothesis regarding specific EEG frequency bands associated with linguistic intrusions. Regarding perceptual imagery reports, some studies indicated right-hemisphere dominance in the imagery-related processes (Whitehouse, 1981; Bryden and Ley, 1983). However, some other studies reported a balanced bihemispheric involvement (Ehrlichman and Barrett, 1983), and even left-hemisphere dominance in the imagery-related processes (Farah et al., 1985; Stinear et al., 2006). We thus could not develop an informed hypothesis regarding hemispheric laterality of hypnagogic imagery.

### HYPOTHESIS 4

We hypothesized that the occurrence of hypnagogic imagery and linguistic intrusions, both of which are unpredictable alterations in the stream of consciousness in a drowsy state of mind, will be associated with changes in the predictability of the EEG signal. One of the recently developed nonlinear measures of EEG signal complexity and predictability is Dimension of Activation (DA; Guillemant et al., 2005). DA has been shown to be sensitive to sleep-triggered EEG changes (Rey et al., 2007), including sleep onset (Magnin et al., 2010): DA value decreases with decreasing complexity of EEG signal that is typical of sleep. By drawing a parallel between the predictability of the contents of consciousness and the predictability of the EEG signal, we hypothesized that the most incongruous hypnagogic reports will be marked by the steepest change of DA in the time window preceding the subjective report.

## MATERIALS AND METHODS

### PARTICIPANT

A case study was carried out with a neurologically and psychiatrically healthy right-handed 90 year-old retired professor of literature (our co-author Massey), who had been observing and self-documenting intrusions for the last 30 years. Massey gave consent and took part in 10 sessions of EEG recording, aiming to collect as many reports of intrusions as possible. The study protocol was approved by the Cambridge Psychology Research Ethics Committee.

### EXPERIMENTAL DESIGN

Massey was instructed to close his eyes, relax, and observe his stream of consciousness, in particular any anomalies in his inner speech. Once a noticeable unpredictable alteration of consciousness occurred, the participant pressed a button with the right hand index finger and verbally reported its content, which was recorded using a digital dictation machine. After transcribing the verbal reports of his experiences (*N* = 191), Massey rated their modality (linguistic, perceptual, or mixed, see **Table 1**) and their degree of incongruence. The scale ranged from 0, meaning that reported linguistic and/or perceptual contents were relatively congruent (low incongruence), to 5, meaning that they were highly incongruent. Reports like “Nonsense word ‘Leeshoe’ came up while I was imagining some sort of overhanging canopy” were common (this one has a mixed modality, and the intruding word is highly incongruent, rated as 5, with respect to the perceptual mentation occurring at that time).

### EEG DEFINITION OF DROWSINESS

The EEG-derived level of drowsiness was visually assessed by two blind judges over 4 s epochs of data (with 15 epochs per single 1 min pre-response period), using the Hori scoring system of 9 stages of sleep onset (Hori et al., 1994). In this system, Stage 1 indicates alpha-dominated relaxation, Stage 9 is marked by complete spindles that coincide with a classical non-rapid eye movement (NREM) Stage 2 sleep, and other stages in-between reflect the gradual progression of sleep onset and the slowing down of dominating EEG frequencies. Reaction times as well as the rate of subjective reports of being asleep are known to increase steadily from Hori Stage 1 to Stage 9 (Hori et al., 1994), corroborating the use of this system to measure the depth of drowsiness. Furthermore, sequential analysis of the progression of Hori stages during uninterrupted transition from wakefulness to sleep confirms the validity of the rank order of these stages (Tanaka et al., 1996).

### EEG ACQUISITION AND ANALYSES

128-channel EEG data, sampled at 250 Hz and referenced to the vertex, were recorded with the Net Amps 300 amplifier (Electrical Geodesics). Channels over forehead, cheeks, and neck were excluded, retaining for analyses 92-channels that covered the scalp. Data pre-processing was carried out using EEGlab toolbox for Matlab (Delorme and Makeig, 2004), including filtering (0.5–45 Hz), average referencing, independent component analysis- based removal of artifacts, and interpolation of noisy channels. After removing reports with the noisy EEG segments, 126 intrusions remained for the analysis of their association with neural dynamics. Following the experiment, Massey explained that due to the difficulty of drifting into a drowsy state of mind while maintaining metacognition it may have sometimes taken him up to 20 s to notice an intrusion, remember the experiences preceding it, and formulate a verbal report before a button press. To account for this broad time window of interest, EEG analyses were carried out using 1-minute segments preceding each button press, which were further subdivided into 60–40 s, 40–20 s, and 20–0 s windows, with 0 indicating the response time. It was assumed that in most of the cases, hypnagogic experiences occurred in the last 20–0 s time window.

Spectral power of theta (3–5 Hz), alpha (7–9 Hz), beta (18–22 Hz), and gamma (35–40 Hz) frequency oscillations was computed using continuous wavelet transform, set from 3 cycles at 1 Hz to 8 cycles at 45 Hz. Frequency bands were defined narrowly to avoid overlap; the alpha band was centered around the dominant 8 Hz frequency, known to decrease with age (Roubicek, 1977). EEG data were analyzed using EEGlab functions and custom-built Matlab scripts. A changing pattern of EEG activity was analyzed by calculating the DA, which reflects the complexity and unpredictability of the EEG signal and has been shown to decrease during sleep onset (Magnin et al., 2010). DA quantifies the complexity of the high-dimensional space occupied by data points consisting of EEG voltage time series by calculating their correlation integral, whose variation is a nonlinear function. As such, DA reflects the amount of temporally correlated information in the EEG signal that depends on the number of EEG frequencies present and their phase relationships (for more details, see Guillemant et al., 2005; Rey et al., 2007; Magnin et al., 2010). Calculation of DA required EEG epochs of 16.384 s length (1024 data points sampled every 16 ms), which were segmented from the middle of their respective time windows.

### STATISTICAL ANALYSES

Given that the Hori system provides an ordinal rather than a continuous measure of drowsiness, non-parametric statistics were applied to analyze Hori-derived data. To compare the peak level of drowsiness in different time windows preceding a button press (60–40 s, 40–20 s, 20–0 s), the maximal Hori stage was selected out of 4 epochs within each time window. Subsequently, the maximal Hori scores were contrasted between the three time-windows using the related-samples Friedman’s analysis of variance (ANOVA) test; pairwise comparisons of any two time-windows were carried out using the Wilcoxon test. Comparisons of maximal Hori scores between different types of reports, i.e., linguistic intrusions vs. perceptual imagery reports, were carried out using the Mann-Whitney test.

Statistical analyses of EEG spectral power, separately for each frequency band, were carried out using the mixed ANOVA test, with Modality (linguistic, perceptual, mixed) as a between-reports factor, and either Time (60–40 s, 40–20 s, 20–0 s) or Hemisphere (left, right) as a within-reports factor. The one-way repeated measures ANOVA test was used to analyze DA values in the three time windows of interest (60–40 s, 40–20 s, 20–0 s). To test a hypothesized association between a shift of DA and an incongruence of hypnagogic reports, DA values were subtracted between 40–20 s to 20–0 s time windows. The obtained difference score as a dependent factor was analyzed by the mixed ANOVA test, with Incongruence (low, high) as a between-reports factor, and electrodes Cluster (anterior, central, posterior) as a within-reports factor. Three clusters were formed by averaging a comparable number of neighboring electrodes (anterior: *N* = 32, central: *N* = 31, occipital: *N* = 29).

In all parametric ANOVA tests, Greenhouse-Geisser corrected degrees of freedom were used to assess the significance of corresponding *F* in a case of the violation of sphericity assumption, as determined by the Mauchly’s Test of Sphericity. When either a main effect or an interaction was significant (*p* < 0.05), independent- or paired-samples *t*-tests were used for *post hoc* comparisons with an unadjusted *p* value. For pairwise comparisons of independent samples, corrected degrees of freedom were used to assess the significance of corresponding *t*, when the Levene’s Test for Equality of Variances indicated an inequality of variances.

Statistical analyses were carried out with the IBM SPSS Statistics (v22) and Matlab software. Only significant main effects, interactions, and pairwise comparisons will be reported in the Results.

## RESULTS

### THE LEVEL OF DROWSINESS PRECEDING HYPNAGOGIC REPORTS

**FIGURE 1:**
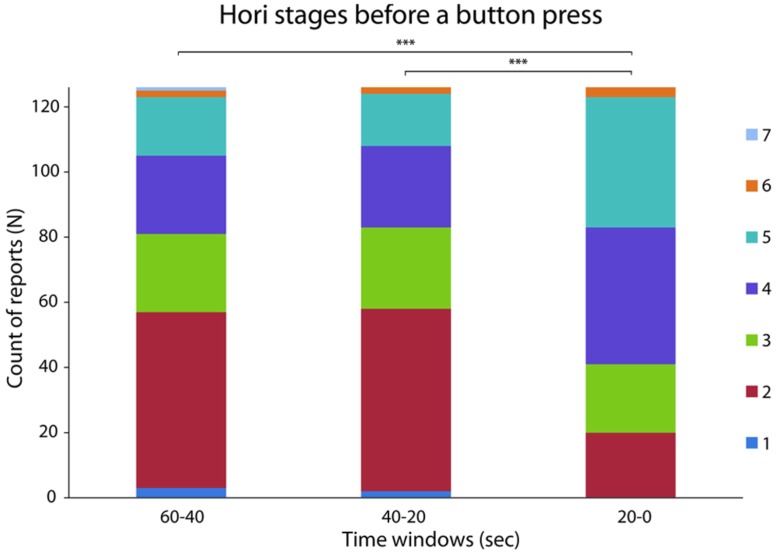
**Distribution of Hori stages in the last 60 s preceding a button press.** The bars indicate the maximal Hori stage from 1 (fully awake) to 7 (advanced drowsiness, late non-rapid eye movement (NREM) Stage 1 sleep) – in the three time windows of interest for each of the 126 intrusions. ***denotes *p* < 0.0005.

**FIGURE 2:**
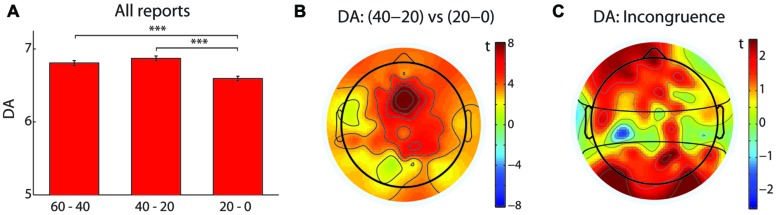
**Dimension of Activation (DA) decrease as a marker of hypnagogic reports. (A)** The red bars indicate DA values, averaged across all electrodes, in three time windows: 60–40 s, 40–20 s, and 20–0 s. All reports were analyzed together (*N* = 126). Error bars indicate the SEM. ***denotes *p* < 0.0005. **(B)** A topographical map of the difference of DA values between 40–20 s and 20–0 s. The color bar denotes a range of *t-*values, with positive values indicating a larger decrease of DA during the last 20 s before the button press. **(C)** A topographical map of the difference of DA decreases between the low incongruence (scored as 0, *N* = 14) and the high incongruence (scored as 5, *N* = 30) reports. Positive *t*-values indicate a larger decrease of DA in the high incongruence compared to the low incongruence reports. The horizontal black curves delineate three electrode clusters (anterior, central, posterior).

When all reports were analyzed together (*N* = 126), a difference in the peak drowsiness, expressed as a maximal Hori stage (Hori et al., 1994), was observed between the three time windows before the button press [*N* = 126, χ_(2)_^2^ = 47.5, *p* < 0.0005, *W* = 0.19; see **Figure 1**]. As expected, drowsiness increased during the last 20–0 s compared to 40–20 s (*Z* = 5.94, *p* < 0.0005, *r* = 0.53) and 60–40 s (*Z* = 5.6, *p* < 0.0005, *r* = 0.5). In particular, an increase in the frequency of Hori stages 4 and 5, and a decrease in the frequency of Hori stage 2 were observed in the last 20 s before a button press, suggesting that the occurrence of unpredictable hypnagogic experiences may depend on a rapid increase of drowsiness. This observation was supported by the analysis of DA, which is known to decrease during sleep onset (Magnin et al., 2010). When analyzed across all reports (*N* = 126), DA values differed between the three time windows [*F*(_2,250_) = 24.53, *p* < 0.0005, $\eta_{p}^{2}$= 0.16; see **Figure 2A**] in that DA was lower during the last 20–0 s compared to 40–20 s [*t*_(125)_ = 6.73, *p* < 0.0005, *d* = 0.61] and 60–40 s [*t*_(125)_ = 4.72, *p* < 0.0005, *d* = 0.41]. A *t* map of DA difference between the 40–20 s and 20–0 s time windows revealed that the steepest DA decrease occurred in the fronto-central electrodes (see **Figure 2B**).

Regarding the modality of subjective reports, the highest Hori stage varied between the three time windows both for the linguistic intrusions [*N* = 44; χ_(2)_^2^ = 8.55; *p* < 0.05; *W* = 0.1] and hypnogogic imagery reports [*N* = 12; χ_(2)_^2^ = 10.05; *p* < 0.05; *W* = 0.42]. Drowsiness increased during the last 20–0 s compared to 40–20 s (linguistic intrusions: *Z* = 3.05, *p* < 0.005, *r* = 0.46; perceptual imagery: *Z* = 2.32, *p* < 0.05, *r* = 0.67) and 60–40 s (linguistic intrusions: *Z* = 2.28, *p* < 0.05, *r* = 0.34; perceptual imagery: *Z* = 2.85, *p* < 0.005, *r* = 0.82), reflecting the general trend of all reports (**Figure 1**). There were no significant differences in the level of drowsiness between linguistic intrusions and hypnagogic imagery reports in any of the three time windows (largest *t* = 1.58), suggesting that similar level of drowsiness is required for both modalities of hypnagogic consciousness to occur.

### MODALITY OF REPORTS: EEG SPECTRAL POWER IN THREE TIME WINDOWS

We aimed to identify EEG markers of linguistic intrusions as opposed to more typical perceptual imagery by contrasting EEG spectral power between these two types of hypnagogic reports. After averaging spectral power across all electrodes, mixed ANOVA tests were carried out separately for each of the EEG frequency bands with Modality (linguistic, perceptual) as a between-reports factor and Time (60–40 s, 40–20 s, 20–0 s) as a within-reports factor.

**FIGURE 3:**
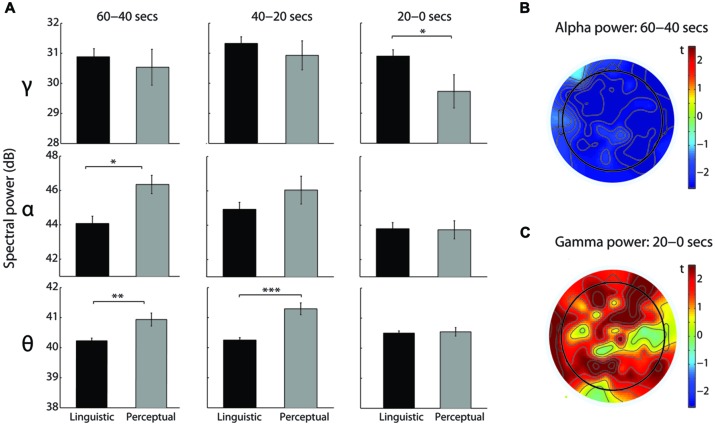
**EEG spectral power differences between linguistic intrusions and perceptual imagery. (A)** Comparison of spectral power in the theta, alpha, and gamma frequency bands between linguistic intrusions (*N* = 44, black) and perceptual imagery (*N* = 12, gray) reports in the three time windows of interest (independent *t*-tests). Spectral power, expressed in decibels (Db), was averaged across all electrodes. Error bars indicate the SEM. *denotes *p* < 0.05, **p < 0.005, and ***p < 0.0005. **(B)** Topographical distribution of the alpha power decrease, expressed as negative *t*-values, in the linguistic intrusions compared to the perceptual imagery reports. **(C)** Topographical distribution of the gamma power increase, expressed as positive *t*-values, in the linguistic intrusions compared to the perceptual imagery reports.

For the theta band, a significant main effect of Modality [*F*_(1,108)_ = 19.81, *p* < 0.0005, $\eta_{p}^{2}$= 0.27], a marginal main effect of Time [*F*_(2,108)_ = 2.89, *p* = 0.06, $\eta_{p}^{2}$= 0.05], and a significant Modality-Time interaction [*F*_(2,108)_ = 10.66, *p* < 0.0005, $\eta_{p}^{2}$= 0.17] were observed. Linguistic intrusions were associated with the lower theta power than perceptual imagery reports in the 60–40 s [*t*_(54)_ = 3.43, *p* < 0.005, *d* = 1.04] and 40–20 s [*t*_(54)_ = 5.7, *p* < 0.0005, *d* = 1.7] time windows (see **Figure 3A**). Furthermore, theta power increased for the linguistic intrusions in the last 20 s compared to 60–40 s [*t*_(43)_ = 2.47, *p* < 0.05, *d* = 0.38] and 40–20 s [*t*_(43)_ = 3.07, *p* < 0.005, *d* = 0.45] time windows. Contrary to this, theta power decreased for the perceptual imagery reports in the last 20 s compared to the 40–20 s time window [*t*_(11)_ = 3.33, *p* < 0.05, *d* = 0.96].

For the alpha band, a significant main effect of Time [*F*_(1.73,93.62)_ = 8.83, *p* < 0.0005, $\eta_{p}^{2}$= 0.14] and a significant Time-Modality interaction [*F*_(1.73,93.62)_ = 3.5, *p* < 0.05, $\eta_{p}^{2}$= 0.06] were observed. Compared to the perceptual imagery reports, linguistic intrusions were associated with the lower alpha power in the 60–40 s time window [*t*_(26.67)_ = 3.33, *p* < 0.005, *d* = 0.95; see **Figure 3A**]. Inspection of a topographical *t* map revealed a generalized decrease of alpha power across all electrodes (see **Figure 3B**). Furthermore, linguistic intrusions were associated with an initial increase of alpha power from 60–40 s to 40–20 s time window [*t*_(43)_ = 2.85, *p* < 0.05, *d* = 0.43], followed up by its decrease from 40–20 s to 20–0 s time window [*t*_(43)_ = 2.74, *p* < 0.05, *d* = 0.41], whereas only a decrease in the last 20 s compared to 60–40 s [*t*_(11)_ = 3.84, *p* < 0.005, *d* = 1.11] and 40–20 s [*t*_(11)_ = 3.12, *p* < 0.05, *d* = 0.9] time windows was observed for the perceptual imagery reports.

Gamma band analysis revealed a significant main effect of Time [*F*_(2,108)_ = 8.57, *p* < 0.0005, $\eta_{p}^{2}$= 0.14] and a marginal Time-Modality interaction [*F*_(2,108)_ = 2.76, *p* = 0.068, $\eta_{p}^{2}$= 0.05]. When compared to the perceptual imagery reports, linguistic intrusions were associated with the higher gamma power in the last 20 s before a button press [*t*_(54)_ = 2.33, *p* < 0.05, *d* = 0.7; see **Figure 3A**]. Visual inspection of topographical heat map showed that the increase of gamma was largest in the frontal, parietal, and left temporal electrodes (see **Figure 3C**). Regarding the three time windows of interest, linguistic intrusions were marked by the gamma power increase from 60–40 s to 40–20 s [*t*_(43)_ = 2.52, *p* < 0.05, *d* = 0.38] and its decrease from 40–20 s to 20–0 s [*t*_(43)_ = 2.56, *p* < 0.05, *d* = 0.39] time window. For the perceptual imagery reports, only a decrease of gamma power was observed from 40–20 s to 20–0 s time window [*t*_(11)_ = 5.56, *p* < 0.0005, *d* = 1.6]. There were no significant differences in the beta frequency band.

### MODALITY OF REPORTS: INTER-HEMISPHERIC ASYMMETRIES OF EEG SPECTRAL POWER

To investigate possible inter-hemispheric asymmetries of EEG spectral power between different modalities of hypnagogic experiences, spectral power was averaged over the three time windows, and mixed ANOVA tests were carried out separately for each EEG frequency band with Modality (perceptual, linguistic, mixed) as a between-reports factor and Hemisphere (left, right) as a within-reports factor.

**FIGURE 4:**
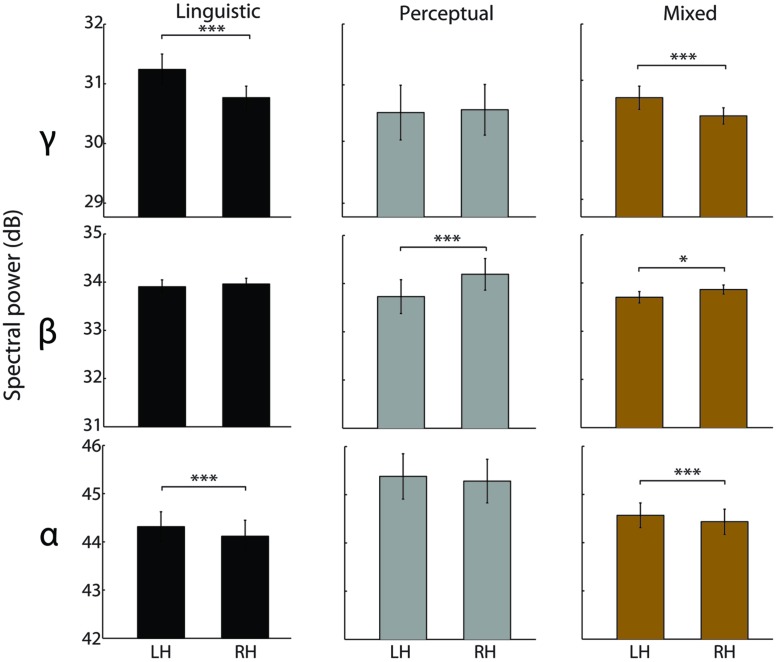
**Inter-hemispheric EEG asymmetries in different types of hypnagogic reports.** Spectral power differences between the left and the right hemisphere electrodes preceding reports of linguistic intrusions (black), perceptual imagery (gray), and mixed content (*N* = 70, olive). The data were averaged over 60–0 s time window. Error bars indicate the SEM. *denotes *p* < 0.05, and ***p < 0.0005.

For the alpha band, a significant main effect of Hemisphere was observed [*F*_(1,123)_ = 10.51, *p* < 0.005, $\eta_{p}^{2}$= 0.08]. Across all reports, the left hemisphere had a higher alpha power than the right hemisphere [*t*_(125)_ = 5.18, *p* < 0.0005, *d* = 0.46]. This inter-hemispheric asymmetry was observed for the linguistic intrusions [*t*_(43)_ = 3.96, *p* < 0.0005, *d* = 0.59] as well as for the mixed reports [*t*_(69)_ = 3.68, *p* < 0.0005, *d* = 0.44], but not for the perceptual imagery reports [*t*_(11)_ = 0.58; see **Figure 4**].

For the beta band, a significant main effect of Hemisphere [*F*_(1,123)_ = 14.19, *p* < 0.0005, $\eta_{p}^{2}$= 0.1] and a marginal interaction Hemisphere-Modality [*F*_(2,123)_ = 2.9, *p* = 0.058, $\eta_{p}^{2}$= 0.05] were observed. Across all reports, the right hemisphere had a higher beta power than the left hemisphere [*t*_(125)_ = 3.24, *p* < 0.005, *d* = 0.28]. This inter-hemispheric pattern was observed for the perceptual imagery [*t*_(11)_ = 7.56, *p* < 0.0005, *d* = 2.19] and mixed reports [*t*_(69)_ = 2.5, *p* < 0.05, *d* = 0.3], but not for the linguistic intrusions [*t*_(43)_ = 0.67; see **Figure 4**].

Finally, a significant main effect of Hemisphere [*F*_(1,123)_ = 8.73, *p* < 0.005, $\eta_{p}^{2}$= 0.07] and a marginal Hemisphere-Modality interaction [*F*_(2,123)_ = , *p* = 0.076, $\eta_{p}^{2}$= 0.04] were observed for the gamma frequency band. Across all reports, the left hemisphere had higher gamma power than the right hemisphere [*t*_(125)_ = 5.18, *p* < 0.0005, *d* = 0.46]. In particular, gamma power was higher in the left hemisphere for the linguistic intrusions [*t*_(43)_ = 3.96, *p* < 0.0005, *d* = 0.59] and mixed reports [*t*_(69)_ = , *p* < 0.0005, *d* = 0.44], whereas no hemispheric asymmetry was observed for the perceptual imagery reports [*t*_(11)_ = 0.58; see **Figure 4**]. No significant differences were observed for the theta frequency band.

### INCONGRUENCE OF SUBJECTIVE REPORTS: DA ANALYSIS

Finally, we hypothesized that the most incongruent reports, i.e., instances of the highest phenomenological unpredictability, will be associated with a change in DA that measures EEG signal predictability. The DA decrease from 40–20 s to 20–0 s time window (see Section 3.1) was analyzed with a mixed ANOVA test, with Incongruence (high, low) as a between-reports factor, and electrode Cluster (anterior, central, posterior) as a within-reports factor. A significant main effect of Cluster [*F*_(2,84)_ = 7.5, *p* < 0.005, $\eta_{p}^{2}$= 0.15], and a significant Cluster-Incongruence interaction [*F*_(2,84)_ = 4.24, *p* < 0.05, $\eta_{p}^{2}$= 0.09] were observed. Pairwise comparisons revealed that highly incongruent reports were associated with a steeper decrease of DA values in the anterior [*t*_(42)_ = 2.06, *p* < 0.05, *d* = 0.71] and posterior [*t*_(41.09)_ = 2.47, *p* < 0.05, *d* = 0.67] electrode clusters, as also visible in a topographical *t* map of a difference of DA decrease between the high incongruence and the low incongruence reports (see **Figure 2C**).

Of the reports with high incongruence scores (*N* = 30), there were eight linguistic intrusions and 22 mixed reports. Among reports with low incongruence scores (*N* = 14), there were five linguistic intrusions and nine mixed reports. Given that the proportion of different modalities of hypnagogic experiences did not differ between incongruence groups [Chi-square with Yate’s correction: χ_(1)_^2^ = 0.07, *p* = 0.8], DA association with incongruence cannot be attributed to EEG differences between linguistic and perceptual contents of hypnagogic consciousness.

## DISCUSSION

The findings of this case study show that the occurrence and the modality of unpredictable experiences of a drowsy mind are strongly and meaningfully associated with the oscillatory patterns of spontaneous EEG, both before and during the occurrence of intrusions. Clinical EEG scoring of the progression of sleep onset (Hori et al., 1994) indicated an increase of drowsiness in the last 20 s before a button press, in particular a higher rate of Hori stages 4 and 5. Interestingly, Germain and Nielsen (2001) also reported a high incidence of hypnagogic imagery after awakening their participants from Hori stages 4 and 5, suggesting that most of the hypnagogic experiences may occur during EEG flattening (Hori stage 4) and the first occurrence of theta ripples (Hori stage 5). In addition to the increase of Hori scores, DA analysis demonstrated a decrease of EEG signal complexity, which typically occurs during sleep onset (Magnin et al., 2010), in a time window preceding linguistic intrusions and perceptual imagery reports. Thus, converging evidence from Hori and DA analyses confirm a subjective impression of our participant Massey as well as sporadic reports in literature that linguistic intrusions do take place in a drowsy state of mind, and that decrease of integration capacity of semantic networks may be yet another feature of hypnagogic consciousness during wakefulness transitions.

As we reported above, we found a decrease of theta and alpha power in the last 20 s compared to 40–20 s time window before perceptual imagery reports, which conceptually replicates a previous report of theta and alpha power decrease during 9–0 s compared to 35–10 s before a report of visual and kinaesthetic imagery during sleep onset (Germain and Nielsen, 2001). While in the present study linguistic intrusions also were associated with a decrease of alpha power in the last 20 s, a significant increase rather than decrease of theta power was observed before a report of linguistic alterations of consciousness. Furthermore, we observed that alpha and gamma power initially increased from 60–40 s to 40–20 s time window before decreasing in the last 20 s for linguistic intrusions, whereas EEG preceding perceptual imagery reports showed no initial increase in power of these frequency bands. Thus, compared to perceptual imagery reports, linguistic intrusions seemed to be associated with more variable temporal dynamics of EEG spectral power in a very broad time window (60 s) preceding a button press. When compared directly, linguistic intrusions were found to be associated with lower theta and alpha, and higher gamma band power than perceptual imagery reports. These findings demonstrate a qualitatively different spectral profile of linguistic intrusions compared to reports of hypnagogic perceptual imagery, providing preliminary electrophysiological evidence of linguistic intrusions being a unique category of hypnagogic experiences, with their own specific neural signatures.

Additionally, linguistic and perceptual intrusions showed distinct patterns of hemispheric asymmetries, further confirming that different modalities of subjective experiences in a drowsy state of mind are associated with distinct modes of neural processing. In particular, linguistic intrusions were associated with higher alpha and gamma power in the left hemisphere compared to the right hemisphere electrodes. Morillon et al. (2010) reported that EEG gamma band power is asymmetrically tuned in auditory regions towards the left hemisphere dominance in response to exogenous audio-visual linguistic stimulation, while our report suggests a similar asymmetry during endogenous linguistic processing. Contrary to this, hypnagogic imagery reports were associated with higher beta power in the right hemisphere compared to the left hemisphere electrodes, which supports the right-hemisphere dominance in mental imagery (Whitehouse, 1981; Bryden and Ley, 1983). Interestingly, mixed content reports containing both linguistic intrusions and perceptual imagery had both higher alpha and gamma power in the left hemisphere, and higher beta power in the right hemisphere electrodes. That is, reports with both linguistic and perceptual contents were associated with spectral EEG markers that were differentially observed in single modality alterations of consciousness.

Analysis of DA decrease between 40–20 s and 20–0 s time windows as a function of the incongruence of hypnagogic experiences revealed an intriguing parallel between rapid changes of predictability patterns in human phenomenology and scalp EEG: the most incongruent reports with unpredictable linguistic content were coupled with decreasing complexity and increasing predictability of the EEG signal. An association between the lower predictability of the contents of consciousness and the higher predictability of EEG time series may seem rather unexpected if not paradoxical. However, a coherent stream of consciousness is likely to depend on or require highly complex information to detect and inhibit irrelevant processes and mental representations, which is needed for the sustainment of a consistent world simulation (Revonsuo, 2006). In situations when EEG complexity decreases and nonlinear activity of the neuronal networks becomes more predictable, a coherent world simulation cannot be further supported and the stream of consciousness may start to disintegrate and fragment, which would eventually lead to the occurrence of unpredictable words or perceptual contents. This rather speculative interpretation of DA findings requires further testing with a larger sample of participants and a broader spectrum of the altered states of consciousness that are marked by high incongruence of internally generated experiences. For instance, bizarreness of dream contents, which is one of the most distinctive features of dream consciousness (Hobson et al., 1987; Mamelak and Hobson, 1989; Revonsuo and Salmivalli, 1995), provides yet another instance of the occurrence of unpredictable alterations of the stream of consciousness that could be probed with the DA analysis.

Unfortunately, the findings of any single case study are of a very limited generalizability. Nevertheless, given the unpredictable occurrence of linguistic intrusions, the unknown rate of their incidence in a wider population, and the large number of reports required for quantitative EEG analyses, we have decided to restrict the present exploratory study to one participant, who had been examining this experience at the phenomenal level for many years, knew what to look for, and how to report it when it happened, and agreed to take part in a long series of EEG sessions. It has been argued previously that highly trained participants should be recruited in studies that require challenging self-monitoring of subtle sleep and dreaming-related contents of consciousness (Nielsen and Stenstrom, 2005), and this approach has been successfully applied in several studies of hypnagogia (e.g., Nielsen, 1995; Stenstrom et al., 2012). As had been hoped, the verbal reports given by Massey during the 10 sessions that were carried out did provide a large quantity of valuable information. The subject was frequently successful in entering a drowsy state and in then saying what had been happening in his mind during the intervals between waking and sleeping. The verbal intrusions reported frequently offered clear-cut instances of striking incongruity between the linguistic components of hypnagogia and those devoted to perceptual imagery.

Another limitation of the present study is the uncertain timing of the occurrence of reported hypnagogic experiences. We have assumed before the analyses that in most cases experiences should have occurred during the last 20 s before a button press, which was supported by several findings, namely an increase of Hori stages and a decrease of DA values in this time window. Yet, a complex cascade of cognitive processes should have preceded and followed hypnagogic experiences, including continuous metacognition and evaluation of the ongoing stream of consciousness, recognition of unpredictable contents, recollection of preceding mental events, decision to respond, and execution of motor command. Thus, it is possible that some of our findings might be more closely related to these or some other mental processes rather than hypnagogic experiences themselves. Nevertheless, assuming that these non-specific cognitive processes should have been approximately similar across all reports, EEG spectral power differences between linguistic intrusions and perceptual imagery reports should arguably reflect a difference in the neural mechanisms of their generation rather than recollection and reporting.

With respect to the timing of the button press, and regarding methodological improvement in future studies, it was not realized until after the study was completed that more specific instructions should have been given to the participant to press the response button as soon as he surmised that a verbal intrusion might have occurred, rather than waiting until afterwards to make sure that a genuine intrusion had actually taken place. If the first procedure had been followed, any error of false alarm could have been easily corrected; but, in the second case, by allowing the subject to wait until he was sure that an intrusion had happened, an additional amount of time could elapse before the decision to report was made, so that correlating the time of the intrusion with the EEG became more difficult.

Despite such limitations, our findings indicate that hypnagogic experiences, in particular linguistic intrusions, and corresponding EEG patterns might be used to study fragmentation of the stream of consciousness in a healthy individual. The occurrence of linguistic intrusions was associated with a steep increase of drowsiness in the last 20 s before a button press. When compared to perceptual imagery, linguistic intrusions were linked to several EEG hemispheric asymmetry measures, in particular a higher alpha and gamma power, and a lower beta power in the left hemisphere. Furthermore, the most incongruent reports with linguistic anomalies were associated with a rapid decrease of DA values, indicating that the occurrence of linguistically incongruous experiences coincides with the rapid change of EEG predictability patterns. Given the exploratory nature and the large number of statistical analyses in this case study, more focused replication studies are needed to determine whether the methodology we have employed is the most effective in establishing an association between the reported EEG markers and linguistic intrusions at sleep onset, but also to evaluate the validity of the preliminary conclusions we have put forward.

## Conflict of Interest Statement

The authors declare that the research was conducted in the absence of any commercial or financial relationships that could be construed as a potential conflict of interest.
